# Supplementary figures and images for: Assessment of dispersion metrics for estimating single-cell transcriptional variability
Source: PLoS Comput Biol. 2026 Mar 2;22(3):e1014030. doi: 10.1371/journal.pcbi.1014030 (PMC12970974; doi:10.1371/journal.pcbi.1014030)

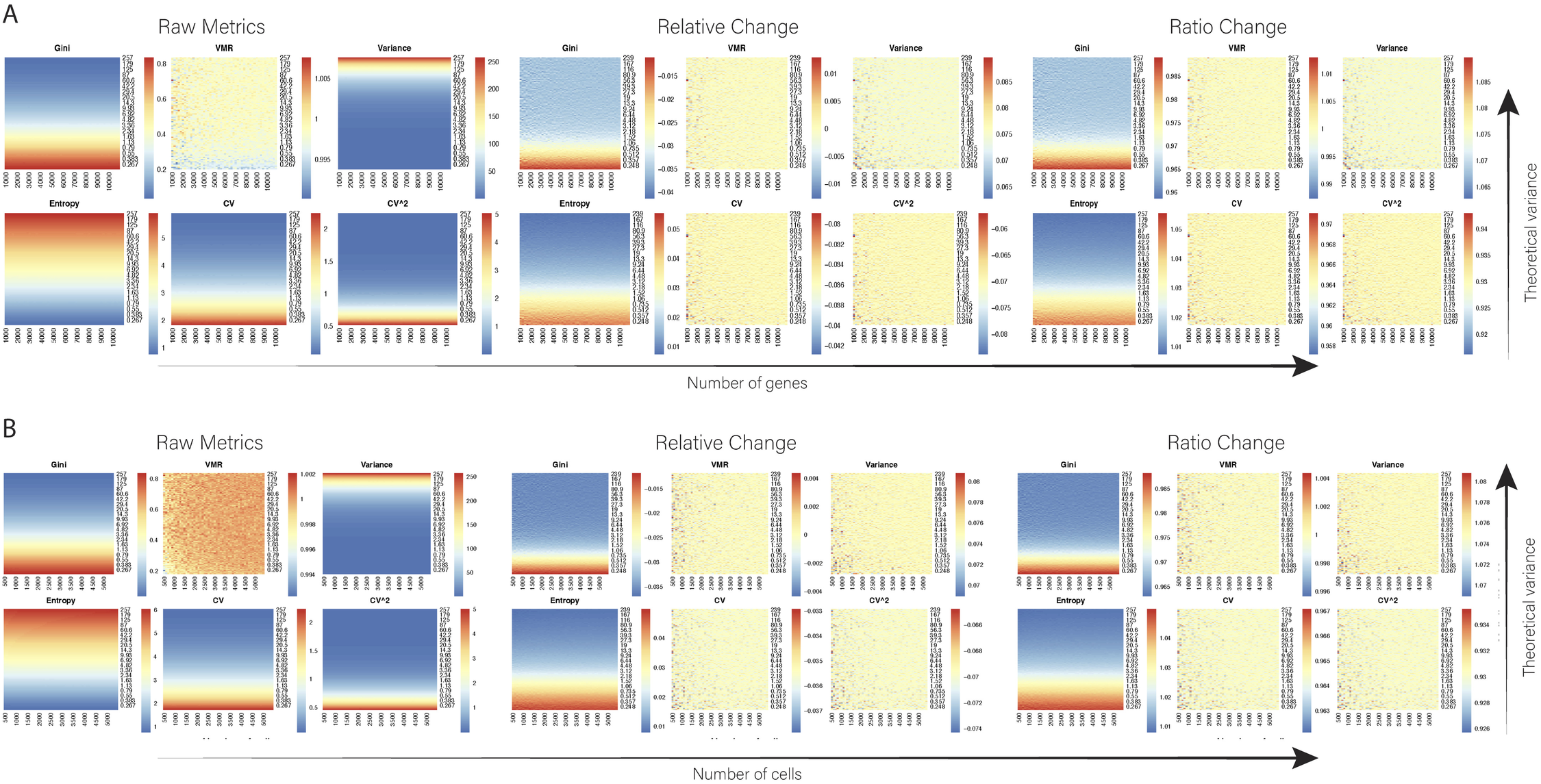

Supplement: S1 Fig — Heatmaps of each metric applied to simulated counts drawn from instances of the Poisson distribution. The dispersion in Poisson (as determined by ƛ) increases across the y-axis. The size of the simulated data increases across the x-axis, as determined by the number of genes (A) or by the number of cells (B) in each counts matrix. (TIF) [file pcbi.1014030.s001.tif]

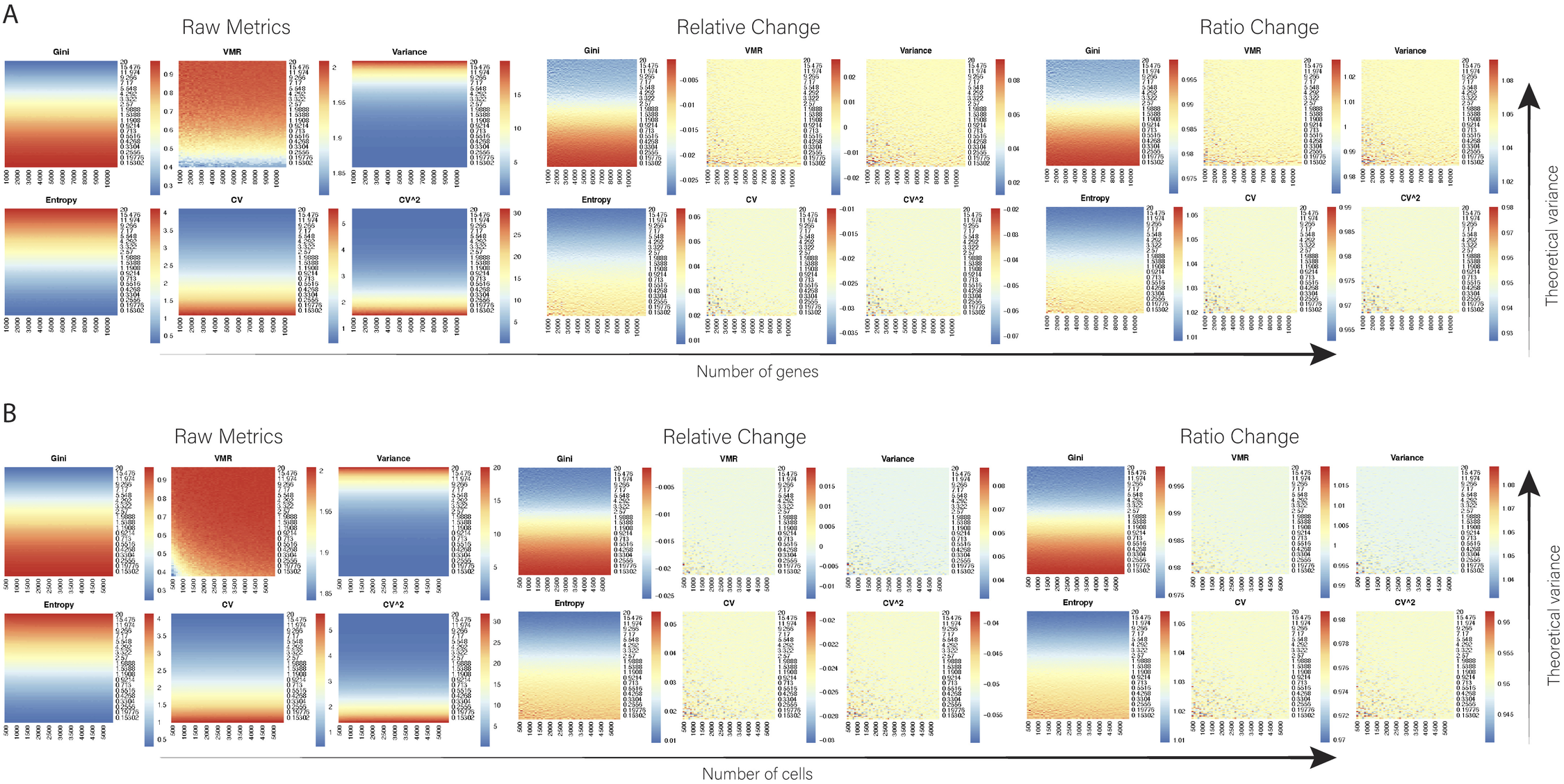

Supplement: S2 Fig — Heatmaps of each metric applied to simulated counts drawn from instances of the negative binomial distribution. The dispersion in the negative binomial (as determined by size parameter r) increases across the y-axis. The size of the simulated data increases across the x-axis, as determined by the number of genes (A) or the by number of cells (B) in each counts matrix. (TIF) [file pcbi.1014030.s002.tif]

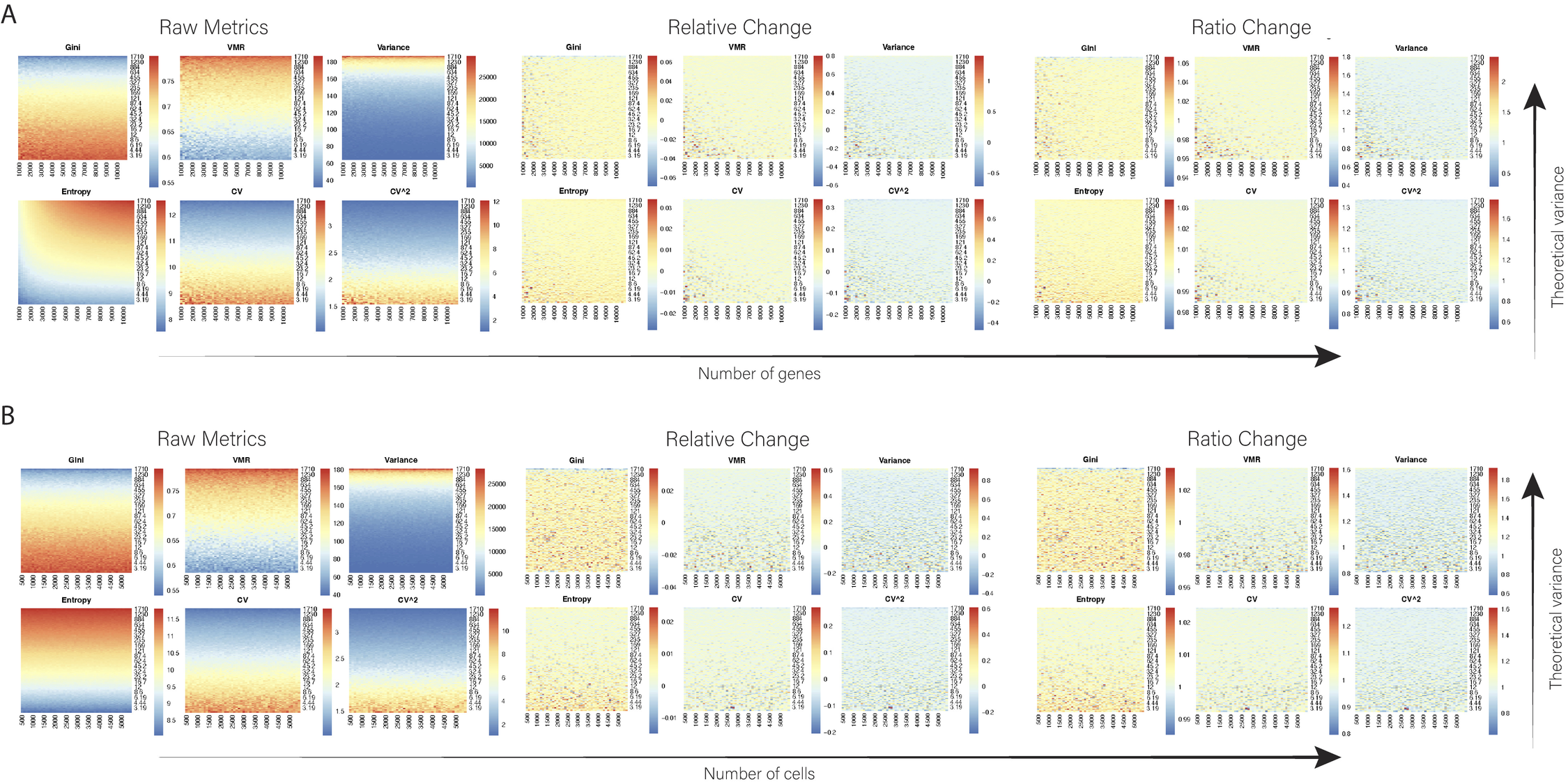

Supplement: S3 Fig — Heatmaps of each metric applied to simulated counts drawn from instances of the beta-Poisson distribution. The dispersion in the beta-Poisson distribution increases across the y-axis, as determined by a scale factor of shape parameter β. The size of the simulated data increases across the x-axis, as determined by the number of genes (A) or by the number of cells (B) in each counts matrix. (TIF) [file pcbi.1014030.s003.tif]

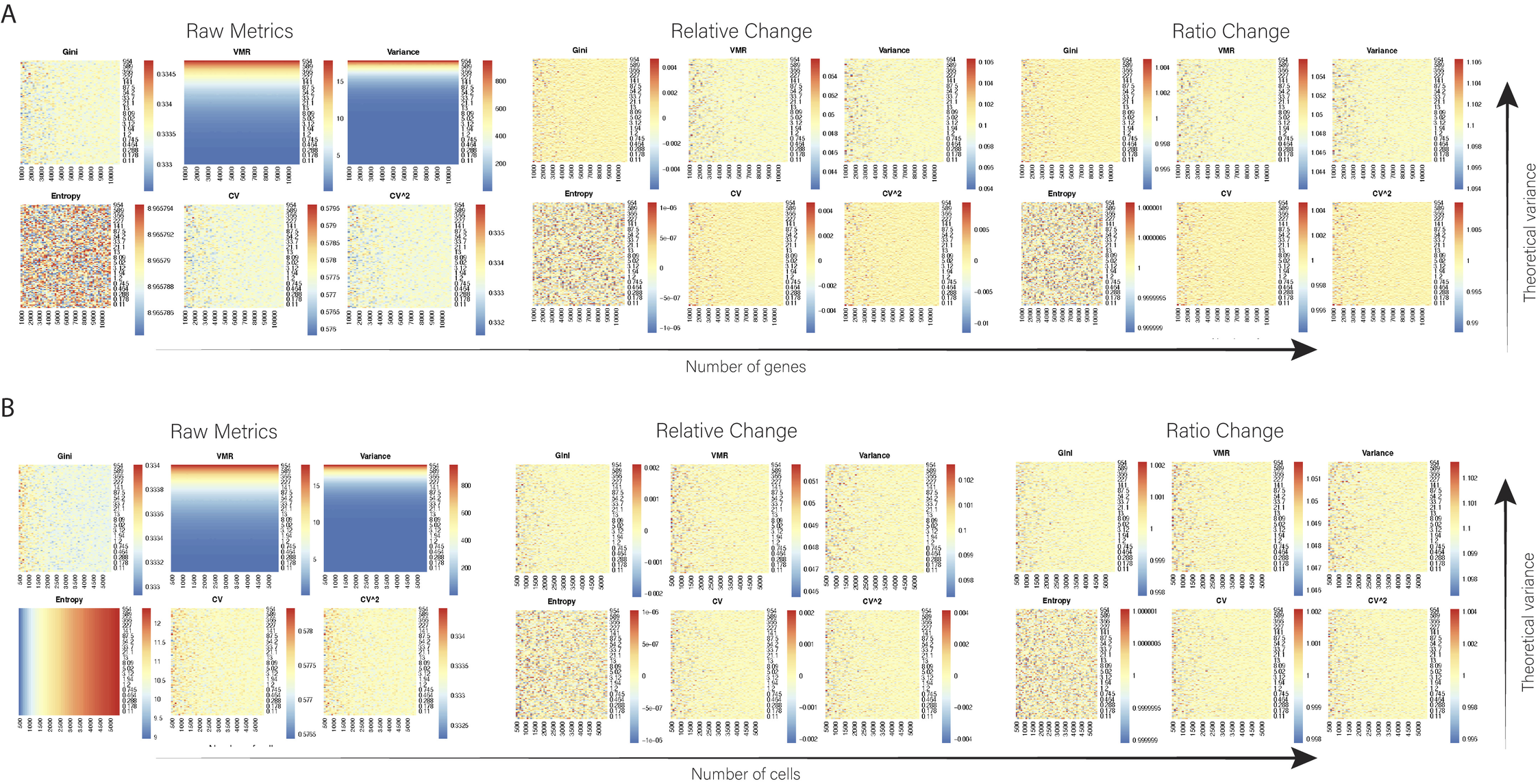

Supplement: S4 Fig — Heatmaps of each metric applied to simulated counts drawn from instances of the uniform distribution. The dispersion in the uniform distribution increases across the y-axis, as determined by maximum value b. The size of the simulated data increases across the x-axis, as determined by the number of genes (A) or by the number of cells (B) in each counts matrix. (TIF) [file pcbi.1014030.s004.tif]

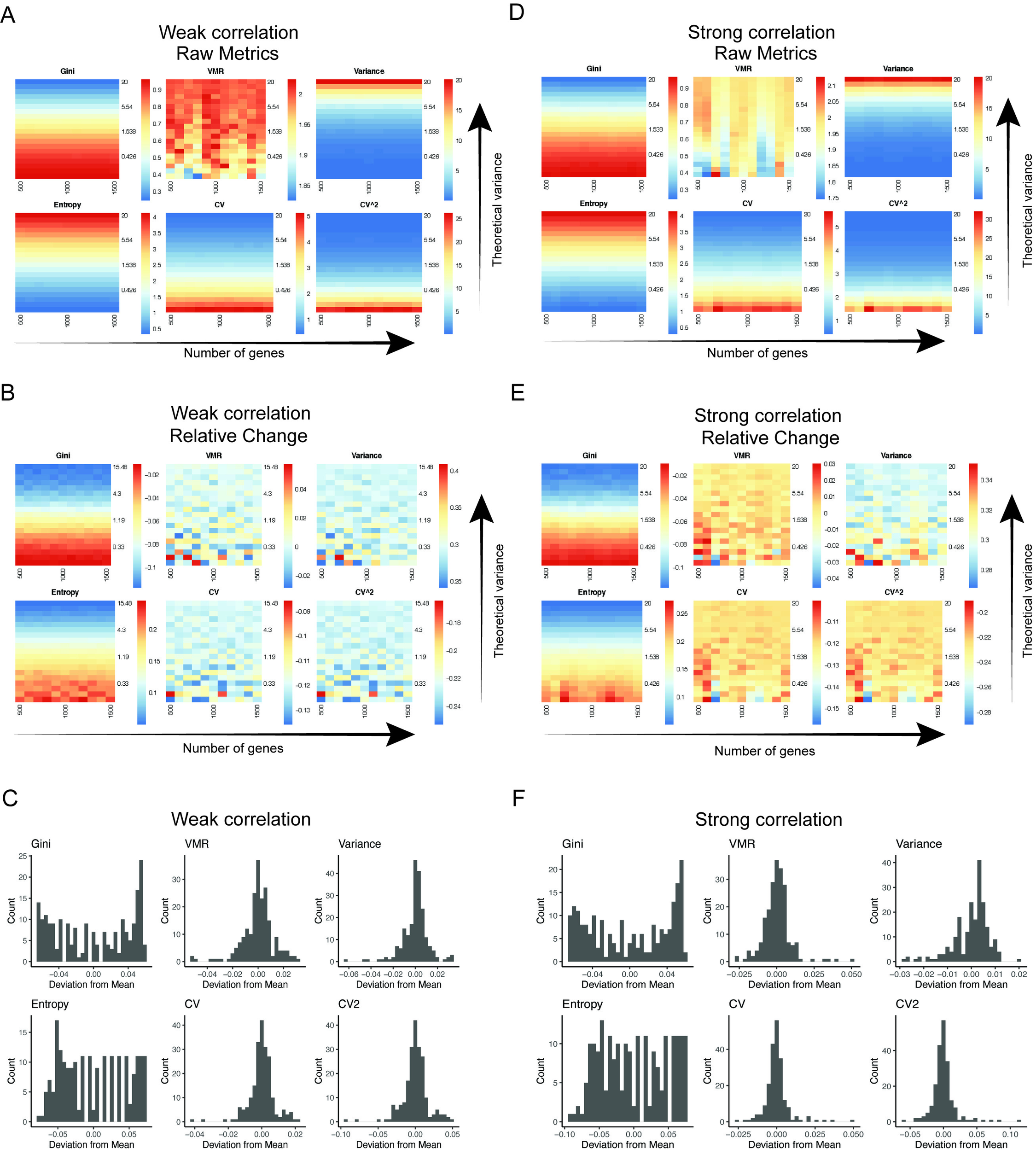

Supplement: S5 Fig — (A) Heatmaps of each metric applied to simulated counts drawn from instances of the negative binomial distribution with weak gene-gene correlation. The dispersion in the sampling distributions increases across the y-axis, as determined by the sigma parameter. The size of the simulated data increases across the x-axis, as determined by the number of genes in each counts matrix. (B) Heatmaps of relative change in each metric applied to counts from (A). (C) Heatmaps of each metric applied to simulated counts drawn from instances of the negative binomial distribution with strong gene-gene correlation. (B) Heatmaps of relative change in each metric as applied to counts from (C). (E, F) Histograms of deviation of relative change from mean relative change for each metric as calculated from the distributions described in (A, B). (TIF) [file pcbi.1014030.s005.tif]

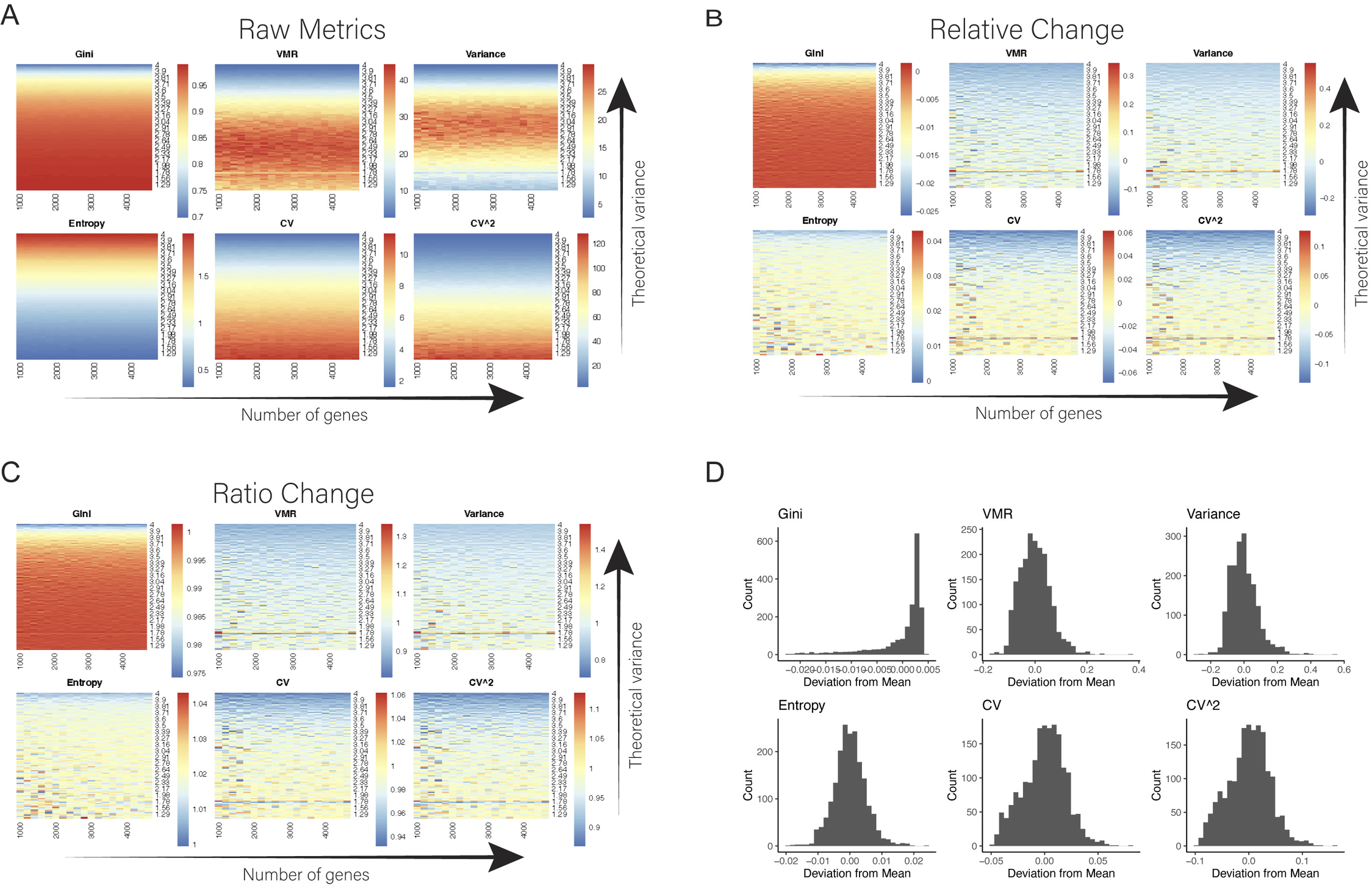

Supplement: S6 Fig — (A) Heatmaps of each metric applied to simulated counts drawn from instances of the Poisson-lognormal distribution. The dispersion in the sampling distributions increases across the y-axis, as determined by the sigma parameter. The size of the simulated data increases across the x-axis, as determined by the number of genes in each counts matrix. (B) Heatmaps of relative change in each metric applied to counts from (A). (C) Histograms of deviation of relative change from mean relative change for each metric as calculated from the distributions described in (A). (TIF) [file pcbi.1014030.s006.tif]

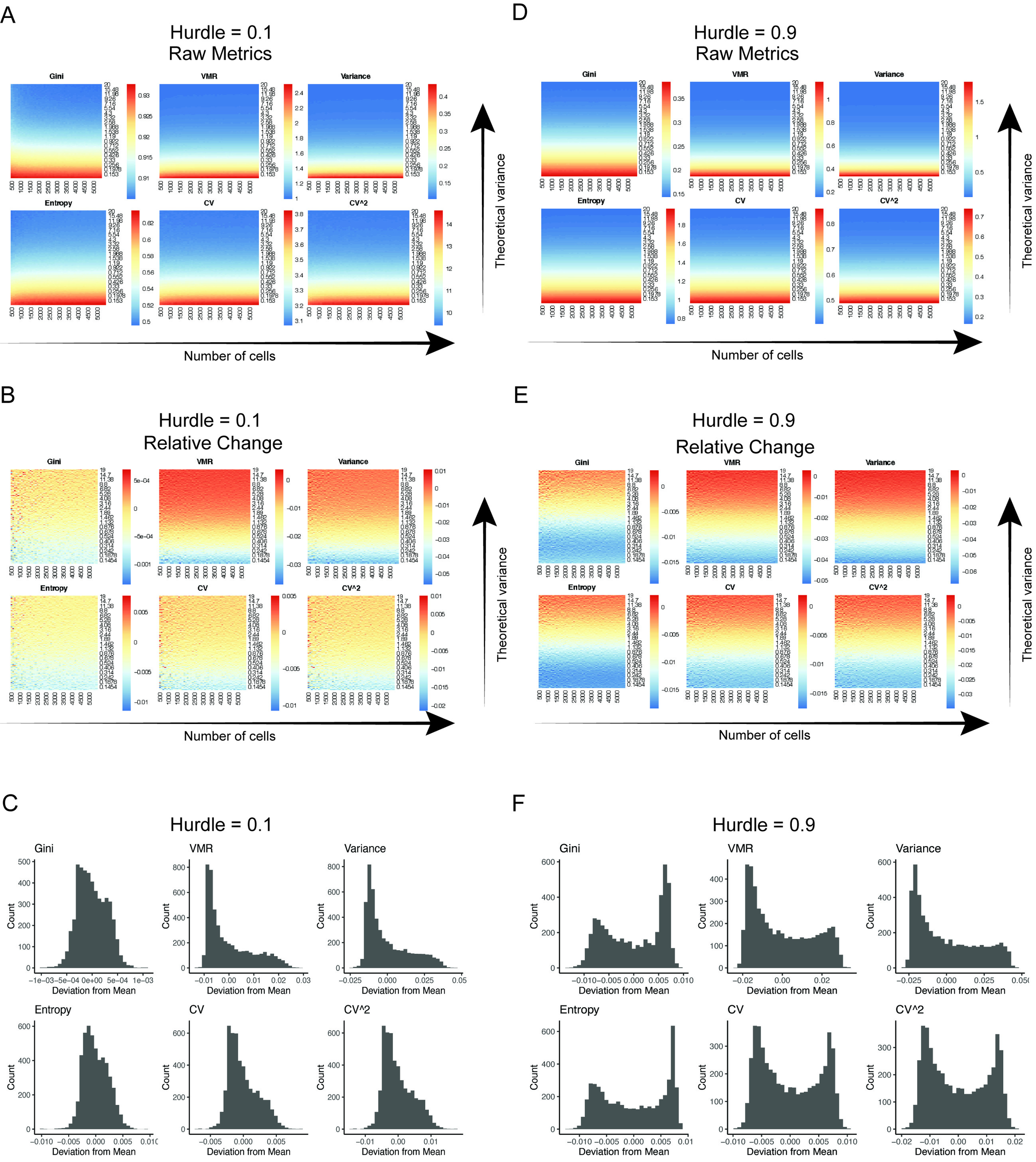

Supplement: S7 Fig — Heatmaps of each metric applied to simulated counts drawn from instances of the hurdle negative binomial distribution with hurdle parameters, which is the probability of a non-zero count, of 0.1 (A) and of 0.9 (D). The dispersion in the sampling distributions increases across the y-axis, as determined by the size parameter. The size of the simulated data increases across the x-axis, as determined by the number of genes in each counts matrix. (B, E) Heatmaps of relative change in each metric applied to counts from (A, D). (C, F) Histograms of deviation of relative change from mean relative change for each metric as calculated from the distributions described in (A, D). (TIF) [file pcbi.1014030.s007.tif]

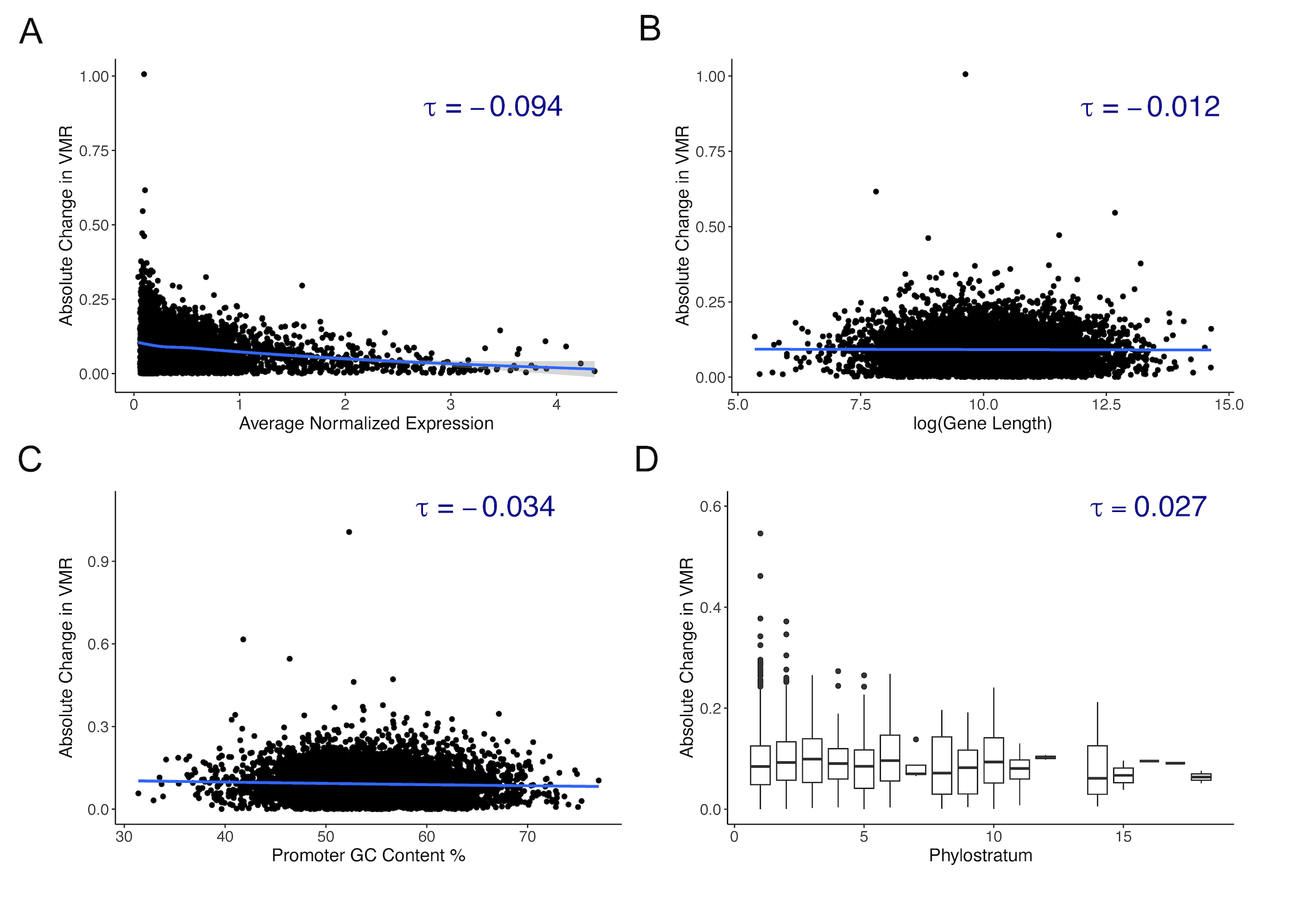

Supplement: S8 Fig — Correlation of absolute change in VMR between control and T21 conditions with (A) mean normalized expression, (B) gene length, (C) promoter GC content, and (D) gene phylostrata in the T21 dataset from Lana-Elola et al. (2024). Kendall’s rank correlation coefficient is shown for each comparison. (TIF) [file pcbi.1014030.s008.tif]

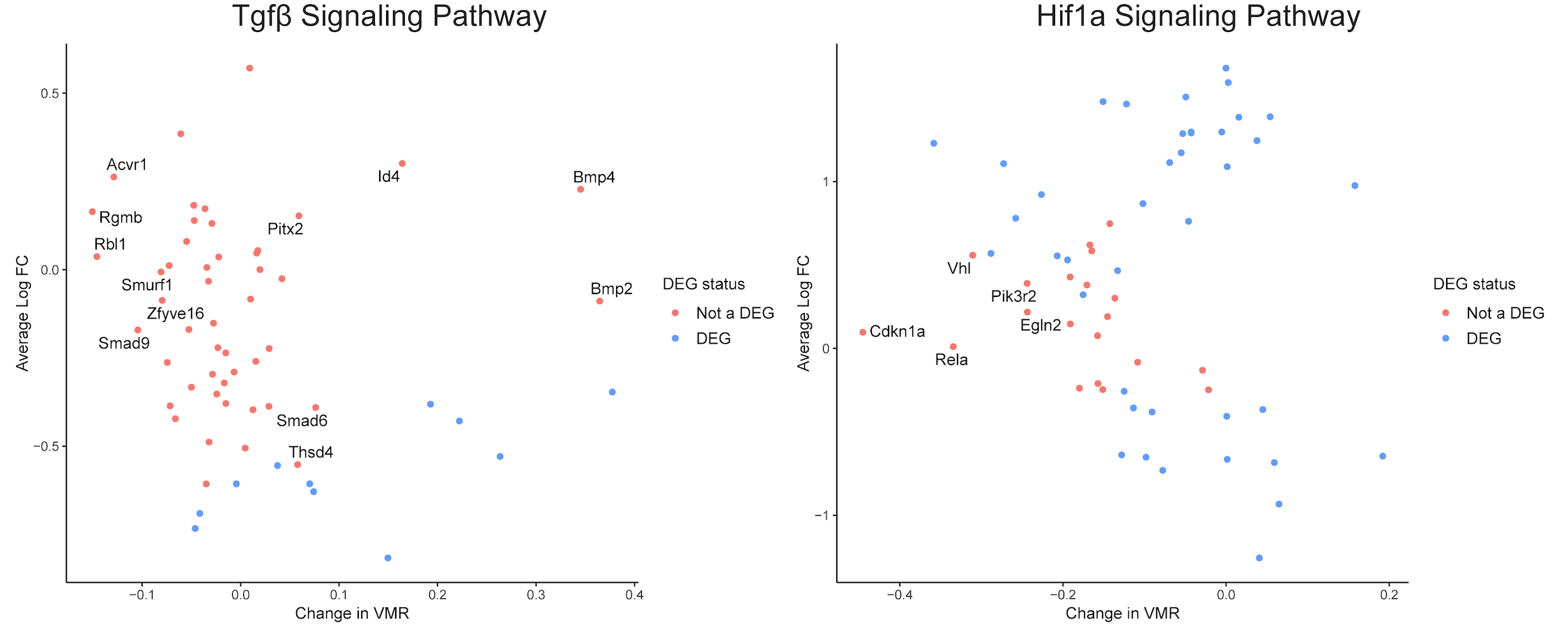

Supplement: S9 Fig — Scatterplots of genes in the Tgfβ and Hif1a signaling pathways plotted by their change in VMR and average log-fold change in the matHG dataset from Manivannan et al. (2022). The Tgfβ signaling pathway was enriched in GSEA of the 1000 genes with largest change in VMR at E9.5. The average log-fold change shown for genes in the Tgfβ signaling pathway is derived from the data at E9.5. Hif1a was enriched in the TF motif enrichment analysis of the 100 genes with largest change in VMR at E11.5. The average log-fold change shown for genes in the Hif1a signaling pathway is derived from the data at E11.5. (TIF) [file pcbi.1014030.s009.tif]

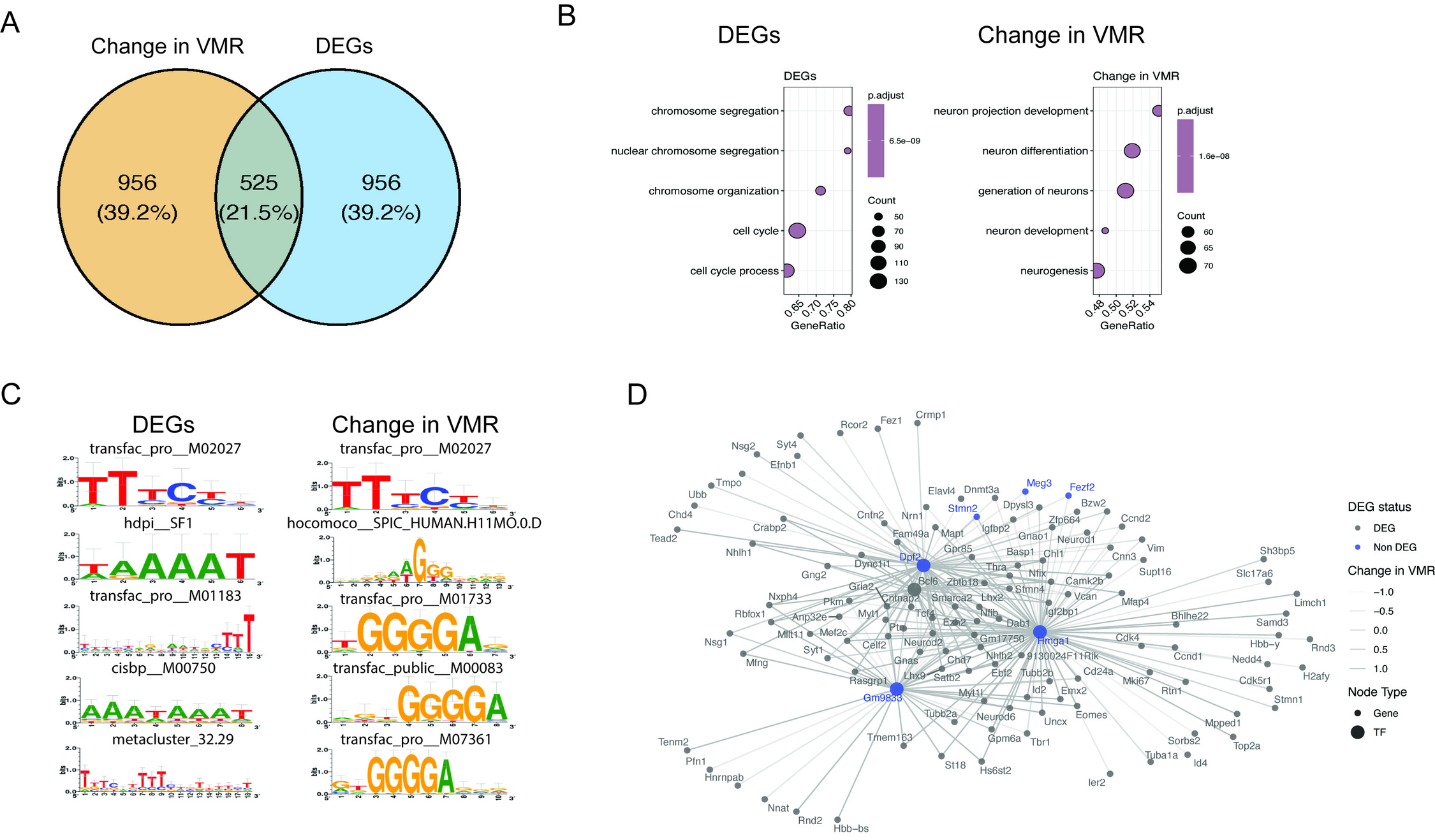

Supplement: S10 Fig — (A) Venn diagram of the 1481 significant DEGs and the 1481 genes with largest absolute change in VMR between E11.5 and at E17.5, including 470 and 711 neurons at E11.5 and E17.5, respectively. Significant DEGs are genes that had an adjusted p-value < 0.05. (B) GSEA using KEGG pathways on the 1000 most significant DEGs, ranked by p-value, and on the 1000 genes with largest absolute change in VMR, ranked by change in VMR. (C) TF motif enrichment was performed on the 100 most significant DEGs and on the 100 genes with largest absolute change in VMR, and the top 5 enriched motifs from each are shown [37]. (D) Dendrogram of TFs enriched among the 100 genes with largest absolute change in VMR and their target genes. Opacity of edge weights is determined by the change in VMR of the genes. DEGs are shown in gray. (TIF) [file pcbi.1014030.s010.tif]
