# Supplementary material for: Assessment of dispersion metrics for estimating single-cell transcriptional variability
Source: PLoS Comput Biol. 2026 Mar 2;22(3):e1014030. doi: 10.1371/journal.pcbi.1014030 (PMC12970974; doi:10.1371/journal.pcbi.1014030)
Supplement: Attachment — Submitted filename: Review of Chen et al.docx [file pcbi.1014030.s013.docx]

**Review of Chen et al, “Assessment of dispersion metrics for estimating single-cell transcriptional variability”**

In this work, Chen et al. performed a computational study to understand the extent to which common measures of dispersion correctly estimate transcriptional variability in counts data such as those generated by single-cell RNA-sequencing (scRNA-seq). The authors primarily simulate count data using different probability models that have been used to model scRNA-seq, before finishing with a study of a published scRNA-seq dataset. The overall message of the work that analysis of single-cell data should involve more than just analysis of changes in mean expression is an important one. However, this is a point that has been made repeatedly in previous works in the literature. Furthermore, I think that the benchmarking could be more thorough and expansive, considering that the authors primarily rely on simulations of probability distributions. **Therefore, I recommend that this manuscript be accepted only after major revisions.**

**Major comments**

- A crucial point that the authors mention is the difficulty in disentangling biological noise from technical noise. To this point, it has been proposed that measurement and expression models should be separated (PMID: 34031584). However, all analysis appears to have been performed on the observed counts without consideration for the contributions to measurement noise due to dropout from sequencing, cDNA construction, batch effects etc, which can distort estimates of transcriptional variability (PMID: 37885177). Therefore, it is not completely clear whether observed dispersion corresponds to genuine biological dispersion.
- In the Introduction, the authors astutely discuss possible sources of transcriptional variability, such as transcriptional bursting, promoter architecture etc. The authors also cite references that describe that the Poisson, Beta-Poisson, and Negative Binomial, arise from assumptions made about gene transcription dynamics (PMID: 22499939). For example, the Beta-Poisson model arises as the steady-state distribution from the classical Telegraph model of regulated, two-state gene transcription. I think it would be worthwhile to mention how different assumptions, mechanistic or statistical, lead to the choices of the different probability models.
- For all of these distributions, the VMRs can be evaluated analytically, provided the parameters are known, while for some of the considered distributions, the entropy also has a closed-form expression. It is not immediately clear, in these cases, what are the benefits of simulating data to evaluate these metrics.
- An implicit assumption by the authors seems to be that the count matrices are generated by independent gene distributions. However, it is known that single-cell data exhibit gene-gene correlations across individual replicates. It would be worth exploring the interplay between gene-gene correlations and transcriptional variability and this would be an application where simulation data would be most useful.
- It is perhaps not surprising that the VMR is the most reliable metric of dispersion. To that point, there have been many methods developed for the task of feature selection from scRNA-seq, several of which are based on VMR. As there have been several studies analyzing the effects of feature selection on downstream analysis tasks including integration, clustering, etc. (PMID: 37598422, 40082610), it would be worth discussing what the study’s conclusions imply for feature selection.
- Another increasingly popular model for overdispersed counts data is the Poisson-lognormal model. It may be worth extending the results to include this model.
- I think there should be a more appropriate version of Figure 3 and its accompanying text to make more concrete connections to scRNA-seq. For example, the Gini index has been proposed to identify rare cell states from scRNA-seq (PMID: 27368803).
- The authors chose a published dataset based on the fact that technical noise due to sequencing would be minimal. However, 10x Genomics is known to capture only about 30% of total mRNA expression and there are other UMI-based sequencing technologies that generate better capture rates of mRNA, such as Smart-seq3 (PMID: 32518404) or VASA-seq (PMID: 35760914). The authors should consider extending their analyses to datasets generated by technologies with greater sequencing depth.
- Considering that the authors are analyzing scRNA-seq with multiple replicates, it would be more appropriate to use pseudobulk methods to infer DEGs, as these account for gene-gene correlations within individual replicates.
- I wonder if there are more principled, statistical tests to evaluate differences in VMR between conditions or datasets. For example, even comparison to ANOVA would be useful. There are also studies that have looked at differences in distributions and transcriptional noise (PMID: 33792651, 36660179, 29959206, 39454576).

**Minor comments**

- Page 19, lines 400–402: “The parameters of the sampling distribution were chosen to simulate real scRNA-seq counts. The mean of scRNA-seq counts is approximately 1, the median is often 0, and the range of the counts can include values in the hundreds.” I think this statement is probably right (although a mean of approximately 1 seems like a strong filter) but some justification in the form of citations or supplementary data is needed.
- Gene names should be italicised.
- For the results in Figure 5, how is “significance” of a DEG measured?
