# Supplementary material for: Assessment of dispersion metrics for estimating single-cell transcriptional variability
Source: PLoS Comput Biol. 2026 Mar 2;22(3):e1014030. doi: 10.1371/journal.pcbi.1014030 (PMC12970974; doi:10.1371/journal.pcbi.1014030)
Supplement: Attachment — Submitted filename: Response to Reviewers.docx [file pcbi.1014030.s015.docx]

Response to Reviewers for PCOMPBIOL-D-25-01049: Assessment of dispersion metrics for estimating single-cell transcriptional variability

**We thank the editors and reviewers for their helpful comments and consideration of our manuscript. The feedback significantly improved the work, and we hope that our revised manuscript is now suitable for publication in PLoS Computational Biology. Please find a point-by-point response to each of the reviewers’ comments below in red.**

Reviewer #1:

In this work, Chen et al. performed a computational study to understand the extent to which common measures of dispersion correctly estimate transcriptional variability in counts data such as those generated by single-cell RNA-sequencing (scRNA-seq). The authors primarily simulate count data using different probability models that have been used to model scRNA-seq, before finishing with a study of a published scRNA-seq dataset. The overall message of the work that analysis of single-cell data should involve more than just analysis of changes in mean expression is an important one. However, this is a point that has been made repeatedly in previous works in the literature. Furthermore, I think that the benchmarking could be more thorough and expansive, considering that the authors primarily rely on simulations of probability distributions. Therefore, I recommend that this manuscript be accepted only after major revisions.

Indeed, analyses beyond mean expression, and those looking at second and third order moments of variance, have been a central focus that remains an important question in the field. While the ability to determine variably expressed genes can be determined using packages such as Seurat or Single Cell Pipeline, our goal was to systematically compare statistical methods for modeling noise using simulations across a diverse parameter space and then to test our assumptions using real data. We have now added more distributions and scenarios to make our benchmarking more comprehensive. In our revised manuscript, we have compared additional statistical methods and extended applications to other single cell data sets that provide a more comprehensive framework for applying these models as well as assumptions underpinning model choice for interpreting variable gene expression across data sets.

Major comments

-	A crucial point that the authors mention is the difficulty in disentangling biological noise from technical noise. To this point, it has been proposed that measurement and expression models should be separated (PMID: 34031584). However, all analysis appears to have been performed on the observed counts without consideration for the contributions to measurement noise due to dropout from sequencing, cDNA construction, batch effects etc, which can distort estimates of transcriptional variability (PMID: 37885177). Therefore, it is not completely clear whether observed dispersion corresponds to genuine biological dispersion.

The reviewer raises an important point. We agree that using observed counts cannot distinguish technical from biological noise. The goal of our work was to systematically assess metrics on observed UMIs to model variance that is interpretable and biologically meaningful. To best account for differences in technical noise due to uneven transcript capture and variable sequencing depth as well as “dropouts”, we compared analyses based on our simulations using different distributions as well as sequencing platforms. Our extensive simulation studies indicate that we are not identifying technical noise and that observed dispersion might indeed correspond to genuine biological dispersion. For instance, we would not expect to identify enriched gene categories or transcription factor motifs within the variably expressed gene categories by chance alone.

Furthermore, our simulation studies captured a broad range of metrics, spanning both parametric (e.g., Fano factor) and nonparametric behavior. Our assumptions are not limited relative to the inherent data distributions that are generating the raw or observed UMI counts as our analyses were performed across linear, nonlinear and heavily parametrized distributions. As the reviewer points out, scRNA-seq datasets are subject to substantial technical noise due to uneven transcript capture, variable sequencing depth across cells, and “dropouts” (undetected transcripts). To that end, our real-world case studies (3 distinct datasets) include data with variable dropout rates, as well as an example with spike in controls. Across each scenario, analysis of transcriptional noise identified potentially biologically meaningful genes and pathways that show variable expression across data sets.

-	In the Introduction, the authors astutely discuss possible sources of transcriptional variability, such as transcriptional bursting, promoter architecture etc. The authors also cite references that describe that the Poisson, Beta-Poisson, and Negative Binomial, arise from assumptions made about gene transcription dynamics (PMID: 22499939). For example, the Beta-Poisson model arises as the steady-state distribution from the classical Telegraph model of regulated, two-state gene transcription. I think it would be worthwhile to mention how different assumptions, mechanistic or statistical, lead to the choices of the different probability models.

Thank you for this suggestion. We have further elaborated on model choices influencing distributions in the main text (Introduction, page 5):

“Other factors that impact model choice include the statistical and mechanical assumptions of each model. For example, the beta-Poisson distribution has been shown to arise from the telegraph model of two-state gene transcription at steady state. On the other hand, the negative binomial distribution captures transcriptional bursts under the telegraph model. Moreover, non-normality in gene expression has been previously considered and implicated in pathophysiologic processes. For instance, a bimodal distribution has been surmised to model genes with distinct on and off transcriptional states, and these switch-like genes have been shown to identify patient subgroups with different survival rates (PMID:18177501). Thus, we sought to systematically compare different noise metrics across a range of distributions that capture various possible scenarios to model the underlying transcriptional output.”

-	For all of these distributions, the VMRs can be evaluated analytically, provided the parameters are known, while for some of the considered distributions, the entropy also has a closed-form expression. It is not immediately clear, in these cases, what are the benefits of simulating data to evaluate these metrics.

As mentioned, the VMR, Gini index, and entropy can be calculated theoretically from a given distribution’s parameters, however, recent work has shown that empirically these metrics can underestimate heavy-tailed and over-dispersed distributions or display non-intuitive behavior for large samples from an infinite-variance distribution (e.g., Milewska *et al.* <https://arxiv.org/abs/2201.12298>; McVinish *et al. Journal of Mathematical Biology91(2)*, 2025). Thus, the goal and value of our simulations are two-fold: (1) to better uncover any unintuitive behavior for outlier or extreme points of a distribution that are not immediately obvious from a closed-form expression, and (2) more directly compare the metrics to each other and assess how they might scale based on the underlying data distribution.

We have added a sentence to the main text reflecting this point: “Empirically, these metrics can underestimate heavy-tailed and over-dispersed distributions; therefore, our simulations sought to better uncover any unintuitive behavior that may not be evident from a closed-form expression, and more directly compare the metrics to each other based on the underlying data distribution.”

-	An implicit assumption by the authors seems to be that the count matrices are generated by independent gene distributions. However, it is known that single-cell data exhibit gene-gene correlations across individual replicates. It would be worth exploring the interplay between gene-gene correlations and transcriptional variability and this would be an application where simulation data would be most useful.

While this is a valid concern, the independence assumption is a useful tool to increase the tractability of one’s modelling framework. We agree, however, that this assumption often does not match reality, particularly when cells are drawn from the same sample or anatomical proximity. To address this point, we simulated counts matrices with varying gene-gene correlations as described in the updated Methods section.

For matrices with weak gene-gene correlation, CV, CV^2^, and VMR exhibited the tightest distributions to noise estimates. As the gene-gene correlation increased, the performance of each of these three metrics improved as well, outperforming the estimates by the Gini index, variance and entropy, which were less robust. This finding is in line with our simulations without gene-gene correlation. When the spread of the normalized values, or relative change, was quantified in matrices simulated from the negative binomial with no gene-gene correlation, CV, CV^2^ and VMR all demonstrated that > 90% of the noise estimates were within 0.5% of the mean relative change in these metrics. These results are now included in the revised manuscript with corresponding text in the Results and Methods sections: “Simulations to assess the impact of gene-gene correlation on metric performance yielded concordant results wherein the Fano factor, CV, and CV^2^ emerged as robust estimators of transcriptional noise across a range of gene-gene correlations (S5 Fig).”

**S5 Fig. Performance of dispersion metrics remains consistent in the presence of simulated gene-gene correlation.** (A) Heatmaps of each metric applied to simulated counts drawn from instances of the negative binomial distribution with weak gene-gene correlation. The dispersion in the sampling distributions increases across the y-axis, as determined by the sigma parameter. The size of the simulated data increases across the x-axis, as determined by the number of genes in each counts matrix. (B) Heatmaps of relative change in each metric applied to counts from (A). (C) Heatmaps of each metric applied to simulated counts drawn from instances of the negative binomial distribution with strong gene-gene correlation. (B) Heatmaps of relative change in each metric as applied to counts from (C). (E, F) Histograms of deviation of relative change from mean relative change for each metric as calculated from the distributions described in (A, B).

-	It is perhaps not surprising that the VMR is the most reliable metric of dispersion. To that point, there have been many methods developed for the task of feature selection from scRNA-seq, several of which are based on VMR. As there have been several studies analyzing the effects of feature selection on downstream analysis tasks including integration, clustering, etc. (PMID: 37598422, 40082610), it would be worth discussing what the study’s conclusions imply for feature selection.

Thank you for this suggestion. We have now added a few sentences addressing this point in our Discussion section:

“Of note, our findings also have implications for feature selection approaches in scRNA-seq analysis. Several existing methods developed for feature selection are based on VMR, and this step in turn affects downstream analysis tasks such as cell type identification and clustering (PMID:37598422, PMID:40082610). Previous work found that highly variable genes influence the quality of scRNA-seq data integration, and our results imply that VMR would likely be a more robust choice for selecting these features, particularly those aimed at data integration for large cell atlas representations.”

-	Another increasingly popular model for overdispersed counts data is the Poisson-lognormal model. It may be worth extending the results to include this model.

Based on the reviewer’s suggestion, we have now included analysis using the Poisson-lognormal model. Specifically, counts matrices were simulated using the R package poilog. The matrices ranged from 1000 to 4800 genes, and all had 500 cells. For each sample size, 100 matrices were simulated from instances of the Poisson-lognormal distribution with varying amounts of dispersion. Six metrics were applied to each count matrix to quantify noise: variance, VMR, Gini index, Shannon entropy, CV, and CV^2^. To evaluate the performance of these metrics across the simulated Poisson lognormal counts, the relative change in each metric as dispersion increased was calculated.

As the theoretical dispersion increased, the Gini index, CV, and CV^2^ decreased, and demonstrated the largest spread of relative change, implying that these metrics may be less robust in this scenario compared to other metrics. Shannon entropy appeared to scale with increasing dispersion in the underlying Poisson-lognormal with the tightest spread of relative change values. Second to entropy, Fano factor and variance also demonstrated tighter spread of noise estimates, suggesting that they may be more robust for overdispersed data compared to metrics such the Gini index, CV, and CV^2^.

We have now added these new data to the manuscript on page 11 (in Results) and in S6 Fig, which reports the results of the Poisson-lognormal simulations.

**S6 Fig. VMR, Variance, CV, and CV^2^ are robust metrics of dispersion on simulated Poisson-lognormal counts.** (A) Heatmaps of each metric applied to simulated counts drawn from instances of the Poisson-lognormal distribution. The dispersion in the sampling distributions increases across the y-axis, as determined by the sigma parameter. The size of the simulated data increases across the x-axis, as determined by the number of genes in each counts matrix. (B) Heatmaps of relative change in each metric applied to counts from (A). (C) Histograms of deviation of relative change from mean relative change for each metric as calculated from the distributions described in (A).

-	I think there should be a more appropriate version of Figure 3 and its accompanying text to make more concrete connections to scRNA-seq. For example, the Gini index has been proposed to identify rare cell states from scRNA-seq (PMID: 27368803).

Our goal in presenting Fig 3 was to show probability density functions and their corresponding Lorenz curves, which demonstrate the paradoxical behavior of the Gini index. Across all our simulations, the Gini index was not a robust metric except in rare scenarios. We are not sure what connections the reviewer may be requesting beyond the empirical paradox represented in the figure. It is plausible that the Gini index might have utility in rare cell type identification, but this topic is beyond the scope of our current work.

-	The authors chose a published dataset based on the fact that technical noise due to sequencing would be minimal. However, 10x Genomics is known to capture only about 30% of total mRNA expression and there are other UMI-based sequencing technologies that generate better capture rates of mRNA, such as Smart-seq3 (PMID: 32518404) or VASA-seq (PMID: 35760914). The authors should consider extending their analyses to datasets generated by technologies with greater sequencing depth.

We initially chose two published datasets based on their homogenous data properties, such as comparable sequencing depth and mean UMI counts per cell. While this limits the technical noise component in the raw UMI counts, we agree that in practicality such datasets are not always available. To further extend our analysis, we have now included analysis of a new time-series dataset that utilized Drop-seq.

In our analysis, we identified 1181 neurons across E11.5 and E17.5 timepoints. Comparing the E11.5 and E17.5 timepoints, we identified 1481 DEGs. We examined the overlap between the DEGs and the 1481 genes with largest absolute change in VMR and found that 525 (21.5%) genes are both DEGs and within the top 1481 genes by change in VMR. We then performed GSEA using GO terms on the top 1000 DEGs by p-value and on the top 1000 genes by change in VMR. The top enriched terms among the VMR genes were neuron development, neuron differentiation, and generation of neurons. In contrast, the top enriched terms among the DEGs were chromosome segregation, cell cycle, and chromosome organization. Neuron development was the 198th enriched GO term among the DEGs, and the neuron differentiation and generation of neurons terms were not enriched at all among DEGs. These analyses were performed using all mouse genes as the background gene set. We also analyzed the top 100 DEGs and top 100 VMR genes for enriched transcription factor (TF) motifs and found that different TF motifs are enriched in these two groups suggesting that different regulatory mechanisms underpin changes in variable genes relative to differentially expressed genes. Notably, ETS TFs which recognize the GGGA motif are critical regulators of neuronal differentiation. Furthermore, these analyses show that analysis of transcriptional noise using the Fano factor is informative across multiple timepoints and on sequencing platforms with different capture rates. These new data are included on page 20 of the revised manuscript and in S10 Fig.

**S10 Fig.** **Transcriptional variability identifies pathways involved in neuronal development.** (A) Venn diagram of the 1481 significant DEGs and the 1481 genes with largest absolute change in VMR between E11.5 and at E17.5, including 470 and 711 neurons at E11.5 and E17.5, respectively. Significant DEGs are genes that had an adjusted p-value < 0.05. (B) GSEA using KEGG pathways on the 1000 most significant DEGs, ranked by p-value, and on the 1000 genes with largest absolute change in VMR, ranked by change in VMR. (C) TF motif enrichment was performed on the 100 most significant DEGs and on the 100 genes with largest absolute change in VMR, and the top 5 enriched motifs from each are shown (PMID:28991892). (D) Dendrogram of TFs enriched among the 100 genes with largest absolute change in VMR and their target genes. Opacity of edge weights is determined by the change in VMR of the genes. DEGs are shown in gray.

-	Considering that the authors are analyzing scRNA-seq with multiple replicates, it would be more appropriate to use pseudobulk methods to infer DEGs, as these account for gene-gene correlations within individual replicates.

We appreciate this suggestion, and as noted above, we further explored the interplay between gene-gene correlations and transcriptional variability in our revised submission through simulations across a range of conditions and data distributions as shown in S5 Fig.

While pseudobulking aggregates gene expression data from groups of cells within the same biological replicate, this approach does increase noise and information loss. The goal of our work was to model the data distribution by taking into account each individual cell. Pseudobulking in this scenario can be misleading as it aggregates expression based on the dataset's cell composition and can deprioritize cell-type-specific information as well as small changes in UMI counts and data distribution.

-	I wonder if there are more principled, statistical tests to evaluate differences in VMR between conditions or datasets. For example, even comparison to ANOVA would be useful. There are also studies that have looked at differences in distributions and transcriptional noise (PMID: 33792651, 36660179, 29959206, 39454576).

The reviewer raises an important point, but respectfully this is beyond the scope of this analysis. As you can imagine, comparing transcripts between different datasets/sequencing modalities requires tackling numerous technical and scale/methodological issues that need to be addressed. This approach is possible, in theory, by using a high-dimensional linear combination of variables or k-tuples of genes between the datasets but is a fundamentally new problem. This would certainly be an area of future investigation, as reflected in the Discussion section: “Future work can be aimed at developing principled statistical tests to evaluate differences in VMR between conditions or datasets to further extend the applicability of modeling noise in scRNA-seq data.”

Minor comments

-	Page 19, lines 400–402: “The parameters of the sampling distribution were chosen to simulate real scRNA-seq counts. The mean of scRNA-seq counts is approximately 1, the median is often 0, and the range of the counts can include values in the hundreds.” I think this statement is probably right (although a mean of approximately 1 seems like a strong filter) but some justification in the form of citations or supplementary data is needed.

The selected range is based on existing literature. We have added the following sentence and reference to the revised manuscript on Page 23. “The mean of scRNA-seq counts is often less than 1 but can also range up to 10 (PMID:31870423).”

-	Gene names should be italicised.

We have now italicized gene names to distinguish them from the proteins.

-	For the results in Figure 5, how is “significance” of a DEG measured?

The significance of a DEG is measured by its adjusted p-value from differential gene expression testing using the Wilcoxon rank sum test. This information has now been added to the figure legend.

Reviewer #2: The manuscript by Chen et al. addresses methods for quantifying variability in scRNA-seq data. Given the rapid expansion of single-cell approaches, this is a timely and important topic, especially considering the wide array of variability metrics currently in use.

Thank you for the support of our work.

The authors compare several measures: Gini index, variance-to-mean ratio (VMR), variance, and Shannon entropy, using simulated scRNA-seq datasets. They show that VMR exhibits particularly favorable properties, including linear scaling with dispersion (Figs. 1–3). This analysis is conducted to a high standard; however, the authors do not include other commonly used metrics, such as the coefficient of variation (CV) [or CV^2]. I believe the data simulations and analytical framework would benefit from a more formal description in the text.

I have concerns regarding the second part of the analysis, which uses previously published data. In particular, the authors use a developmental dataset to explore relationships between VMR changes and sequence features (Fig. 4 and similar analysis in Fig. S6 for analysis of T21). While they report limited correlations, I wonder whether this is because VMR changes between the two conditions are, in fact, minimal. Prior studies have shown that VMR remains relatively stable across conditions for individual genes, at least in the context of the innate immune response (see Bagnall et al., 2020 [<https://doi.org/10.1016/j.cels.2020.08.007>] and Alachkar et al., 2023 [<https://doi.org/10.3389/fmolb.2023.1176107>]). See specific comments below.

We agree that VMR has been used successfully to assess gene-level variability with biological implications in the context of innate immunity, as the reviewer mentions. We anticipate that variable genes are context-dependent, and the magnitude of dispersion will likely be different across scenarios. We have now added a sentence to the Discussion section on page 22 reflecting this point:

“VMR has also been used successfully to assess gene-level variability with biological implications in the context of innate immunity (PMID:32918862, PMID:37441161). We anticipate that variable genes are context-dependent, and the magnitude of dispersion will likely be different across scenarios.”

In Figure 5, the authors demonstrate that differentially expressed genes (DEGs) and high-VMR genes are largely non-overlapping in the developmental dataset. This observation supports the idea that basally expressed genes, that is those not induced in a given context, can nonetheless be highly variable. The authors suggest that VMR analysis could provide novel insights beyond standard DEG approaches. However, they fall short of demonstrating specific advantages in the context of the data analyzed. Notably, the identification of highly variable genes (HVGs) is already a standard and essential step in 10x Genomics scRNA-seq workflows, including in tools like Seurat (R), Scanpy (Python), Cell Ranger, and Bioconductor packages such as scran and scater. I encourage the authors to include additional, more complex datasets encompassing multiple conditions. This would allow for a more rigorous assessment of the utility of VMR-based analysis and strengthen the broader applicability of their conclusions.

We appreciate the reviewer’s comment. However, we would like to clarify that the goals of this work were two-fold. First, we aimed to systematically assess commonly used “noise” metrics across a representative set of statistical models to determine which statistical measure of noise might be best suited for single cell data depending on the underlying data distribution. Second, we wanted to highlight that harnessing these noise metrics can provide a new lens to look at gene expression – one that yields insights that traditional differential testing approaches might miss. We use a diverse set of datasets, across different sequencing platforms, time points, and data distributions, to showcase both our goals. Wet-lab validation for any of the computationally identified targets is outside the scope of our current work.

Additionally, we would like to point out that Seurat uses VMR to identify variable genes for dimensionality reduction, and not for identifying potentially biologically meaningful genes that show variable expression across data sets.

The manuscript is generally well written, but some sections would benefit from further clarification- please see specific comments below.

Major comments:
1) Fig 1: line 400- Some aspects of the data simulations are not clear. The numerical scheme should be described more formally. The authors state for one of the distributions: “For the Poisson distribution, we varied the lambda parameter λ from 0.2 to 2278, where λ increased exponentially.” Similar scheme is described for other distributions. There is varying number of cells and genes per individual cells in simulation. The authors must simulate the range of expression between different genes, i.e., low expressing vs. high expressing. It seems that each gene per individual cell is sampled independently from the reference distribution. The number of different levels of lambda (e.g., for Poisson) seems to be fixed to 10 for all distributions. Should simulation of larger number of genes require a finer gradation of the lambda parameter (and corresponding parameters for other sampling distributions)? After all 10 seems to be quite low comparing to number of genes samples (up to 10,000). The simulation scheme should be also represented in more details in the Figure 1.

The following text has been added to the manuscript on page 9 to clarify the numerical scheme of the simulations:

“For each distribution, we generated multiple instances of the same distribution with differing levels of dispersion (Fig 1A). From each instance, we simulated multiple counts matrices of varying sizes. Counts for each gene within a given counts matrix were independently simulated. All genes within a given counts matrix were simulated from the same distribution, with the same parameterization and dispersion. More specifically, for each family of distributions (i.e., Poisson, negative binomial, etc.), we generated 100 instances of the distribution with increasing levels of dispersion. For each instance of a distribution, we simulated 50 counts matrices, which all had the same number of genes but varying numbers of cells. All genes within a given matrix were simulated from the same distribution instance (i.e., same level of dispersion) and assuming no gene-gene correlation.”

2) Fig. 2. Authors should provide some quantification (beyond a simple drawing of the heat map) as they claim that VMR is most sensitive. Gini appear to exhibit similar to VMR linear scaling of sensitivity, although as author describe with an inverse relationship between index and dispersion. What about other measures, in particular CV (or CV^2) typically used for the quantification of noise- I presume the scaling will not be linear.

We have now included CV and CV^2^ to our simulations. In the Poisson and negative binomial distributions, CV and CV^2^ decreased as dispersion in the counts matrices increased. This is undesirable behavior for a metric of dispersion. However, normalized, or relative change, CV values were very consistent. As the increase in theoretical dispersion is fixed, consistent normalized metric values indicate that the metric scales well with the theoretical dispersion.

As previously discussed, it is important to note that measuring the relative change in CV values may be unreliable as it is not a linear variable. Since many of the simulations involve the Poisson distribution, where mean and variance are inherently connected, they may be impacted by this quality of the CV.

This additional analysis is included on pages 11-12 and in the Discussion, as well as in a new version of Fig 2.

**Fig 2. VMR is most sensitive to changes in dispersion**. (A) Heatmaps of each metric applied to simulated counts drawn from instances of the negative binomial distribution. The dispersion in the sampling distributions increases across the y-axis, as determined by the size parameter r for the negative binomial. The size of the simulated data increases across the x-axis, as determined by the number of cells in each counts matrix. (B) Heatmaps of relative change in each metric applied to counts from (A). (C) Histograms of deviation of relative change from mean relative change for each metric as calculated from the distributions described in (A). (D - F) Same as above for counts simulated from the beta-Poisson distribution. The theoretical variance increases as determined by a scaling factor on the β parameter.

3) Figure 4 presents an analysis that I find somewhat oversimplified. The relationship between gene expression variability and sequence features is a complex topic that has already been explored in prior literature, particularly in an evolutionary context. Notably, Hagai et al. (2018) [<https://doi.org/10.1038/s41586-018-0657-2>] demonstrated that variability across species and contexts is linked to features such as promoter architecture and coding sequence properties. These findings highlight that gene regulatory architecture and evolutionary pressures can shape the intrinsic variability of gene expression.

Authors report lack of correlation between VMR and features. However, I find that the changes in VMR are overall very small, which might contribute to the lack of statistical power.

Given a fold change (FF) in VMR defined as (based on Table S1):

FF = abs (change in VMR)/max(VMR control, VMR MatHG)

Out of 881 genes (E9.5 change in VMR), there is only ~10% (950) of genes exhibits FF greater than 0.2 (20%), and 2% (184) genes greater than 0.3.

For the E11.5, there is 7% of genes with FF>0.3, and 35% with FF>0.2.

Therefore, the actual changes of VMR are very small overall. This is also the case of the second dataset analysed by authors (Fig. S6).

I refer the authors to work by Bagnall et al and Alachkar et al, who showed that VMR remains invariant across different environmental conditions, while the relative changes of variance and mean in mRNA/read counts can be related to parameters of transcriptional bursting. This work suggests that no changes in VMR across conditions are expected, which is in a broad agreement with what authors are finding.

The dataset presented by authors includes only two-time-points (as far as I understand), but perhaps it is worth to test if the differences between VMRs are statistically significant in the dataset present. Perhaps analysis of the data grouped according to range of VMR values (similar to analyses performed by Hagai et al.) will show stronger trends. I would also encourage authors to look at other datasets which include more experimental conditions to extend this analysis beyond 2 time-points. The authors' approach in Figure 4 could be strengthened by incorporating a more nuanced interpretation of sequence-feature relationships.

We agree that VMR has been used successfully to assess gene-level variability with biological implications, as the reviewer mentions. We anticipate that variable genes are context-dependent, and the magnitude of dispersion will likely be altered across scenarios. We have now added a sentence to the Discussion section on page 22 reflecting this point:

“VMR has been applied to assess gene-level variability with biological implications in the context of innate immunity (PMID:32918862, PMID:37441161). We anticipate that variable genes are context-dependent, and the magnitude of dispersion will likely be different across scenarios.”

We have no *a priori* assumption that VMR will or will not change across conditions, based on limited statistical benchmarking done in this field beyond a few select studies as the reviewer kindly points out. Our goal in this work was to systematically compare noise metrics across various data distributions and single cell scenarios to determine which metric may be the most robust and scale-invariant. To that end, for most distributions, VMR is a reasonable, robust choice for modeling transcriptional noise. While we agree that the changes are subtle, we would like to respectfully point out that in this work, we are not making claims about the magnitude of change in VMR that may or may not be expected across conditions. The goal of this work was to systematically assess different noise metrics, and we observe that even subtle changes can be detected in a systematic application of certain metrics and potentially used for biological/functional inference.

Additionally, as noted below, we have now added analysis on another published dataset with multiple time points generated using Drop-seq, where VMR seems to again demonstrate biological utility.

4) In Figure 5, the authors essentially argue that basally (or constitutively) expressed genes in a given context can be highly variable, often more variable than differentially expressed genes (DEGs). While they show that the sets of high-VMR genes and DEGs are largely non-overlapping, this observation is not novel. It is well established that basally expressed genes can display substantial variability across single cells. Moreover, the finding that DEGs are less variable is expected: these genes often respond to strong, uniform signals (e.g., cytokines or developmental cues), resulting in synchronized transcriptional responses across cells. In contrast, variability in basal gene expression may reflect asynchronous states, local microenvironmental influences, or stochastic differences in transcription factor activity, all of which can lead to high cell-to-cell variability, even in the absence of external perturbation. While this finding confirms existing knowledge, the authors’ analysis lacks deeper functional insight. I would strongly recommend including more complex or richer datasets, ideally encompassing multiple conditions or cell types to more convincingly demonstrate the added value of VMR-based analysis. Simply showing that VMR and DEG analyses yield different sets of genes is not sufficient. A clearer demonstration of the functional relevance of high-VMR genes would strengthen the manuscript and better support the claim that VMR captures biologically meaningful variability beyond standard DEG approaches.

The reviewer makes an important point. We would like to clarify that our goal in this work was to systematically compare noise metrics across various data distributions and single cell scenarios to determine which metric may be the most robust and scale-invariant. In this work, the high variance of basally expressed genes recapitulates prior findings, further corroborating the biological utility of this work. To further incorporate a more complex dataset from a different sequencing platform than the ones used in our original submission, we have now added analysis on another published dataset with multiple time points generated using Drop-seq.

It is also worth mentioning that in this submission for PLoS Computational Biology*,* our goal was not focused on new biological discoveries, but to systematically benchmark statistical methods for assessment of biological noise in single cell data. We do not make any functional claims about basally expressed genes as these are outside the scope of this study. Furthermore, we show that enriched gene categories and enriched TF motifs differ between variable and DEGs which may lead to additional biological insights. We are best suited to show this in silico through examples, and manipulation in mouse studies or through wet lab experimentation is beyond the scope of this work.

Minor comments:

31: “the Gini index displayed paradoxical behavior” please spell out what you mean here – the abstract should capture some details of the main findings

We have now clarified this point in the text. For the Gini index, a value of 0 indicates perfect equality, and a value of 1 indicates maximal inequality. As a result, we initially hypothesized that the Gini index would increase as dispersion increased. In contrast, the simulations showed that as the theoretical variance increased, the Gini index decreased. This behavior initially seemed paradoxical to the definition of the Gini index. For additional results and discussion of this point, please see Results subsection: Gini index decreases as variability in scRNA counts increases.

57: “Single-cell RNA sequencing (scRNA-seq) approaches have enabled the study of gene expression at high resolution. “ So does bulk-cell RNA-seq, be more specific.

We have revised this sentence in the Introduction as follows: “Single-cell RNA sequencing (scRNA-seq) approaches have enabled the study of gene expression in individual cells to better appreciate stochastic biological processes and precisely map cell states.”

127: ”we simulated counts matrices from 100 instances with differing levels of dispersion” Please clarify instances as described in the Methods section? This phrase also appears in the Methods (line 397).

The following has been added to the manuscript to clarify numerical scheme of the simulations:

“For each distribution, we generated multiple instances of the same distribution with differing levels of dispersion (Fig 1A). From each instance, we simulated multiple counts matrices of varying sizes. Counts for each gene within a given counts matrix were independently simulated. All genes within a given counts matrix were simulated from the same distribution, with the same parameterization and dispersion. More specifically, for each family of distributions (i.e., Poisson, negative binomial, etc.), we generated 100 instances of the distribution with increasing levels of dispersion. For each instance of a distribution, we simulated 50 counts matrices, which all had the same number of genes but varying numbers of cells. All genes within a given matrix were simulated from the same distribution instance (i.e., same level of dispersion) and assuming no gene-gene correlation.”

137/139: “Schema of/for” should be “Schematic of…”

Thank you, we have corrected this typo.

388 : Methods: in places unclear, for example: “For example, the biological intuition behind Poisson(λ = k) is that it is the probability k UMIs align at a given loci assuming a Poisson distribution.” should be “For example, the Poisson(λ) distribution models the probability that k UMIs align at a given locus, assuming UMI counts follow a Poisson process.”

Thank you for this clarification. We have revised this sentence according to the reviewer’s suggestion.

Fig S1-4 captions: I am guessing that the numbers 0.573 to 83.8 correspond to Poisson parameters (equivalent of the variance) across genes (in the Raw data on the left panel). Corresponding numbers for the normalised data appear to be substantially different, especially in the case of Poisson, please explain how are they calculated?

The x-axis in the heatmaps showing the raw metric values are the parameter values from the distribution that was used to generate the counts. The x-axis in the heatmaps showing the normalized values of the metrics (relative change, ratio change) is depicting the theoretical variance of the distributions. The figures have now been updated so that the x-axis is always showing the theoretical variance.

Figs S1 and S3 seem to be identical.

We have now updated the manuscript to reflect the updated supplementary figures.

144: Why the differences in the scale-invariance in Entropy for different sampling distributions, please provide some insights.

Previous work by Frank and Smith (Entropy 2010, 12, 289-303; <https://doi.org/10.3390/e12030289>) and Nieves *et al.* (PMID: 20867614) found that while a standard Gaussian distribution's differential entropy changes with scale (i.e., it is not scale-invariant), the distribution resulting from a maximum entropy principle with certain constraints can be scale-invariant, often manifesting as a power law or a linear-log exponential distribution.

We have now added the following to the Discussion section: “The differences in the scale-invariance in entropy for different sampling distributions have been previously described for certain distributions. These likely result from differences in linear versus logarithmic scaling of a distribution, and how the closed-form function of entropy relates to its dependence on the first and second central moments of a distribution.”

153: Figs S1, S2 and S4 are referred out of order in the text (i.e., after the S3 and S5).

We have revised the numbering of these supplemental figures to follow the presentation order.

176: The Figure 3A refers to negative binomial, yet the discussion following the initial stamen is related to Poisson: “An intuition for this paradoxical behavior can be observed from the shapes of assessed distributions (Fig 3A). For example, in the Poisson distribution, the Gini index indicates highest noise when the mean and variance of the distribution are less than 1, which corresponds to data with a high proportion of zeros. When the mean and variance of the Poisson are greater than 1…” Of note, mean and variance of Poisson is always equal to lambda, please make sure that you clarify the difference between the sampling distribution and the distribution obtained in the simulated data.

Thank you for pointing this out and we apologize for the confusion. Fig 3 indeed refers to the negative binomial distribution. We are using these plots to highlight the paradoxical behavior of the Gini index and have amended the text to reflect the same. As the reviewer points out, Fig 3A is plotting the probability density functions of negative binomial distributions and 3B shows the corresponding Lorenz curves.

190-199- It would be useful to use a more relevant intuitive example, perhaps referring to read counts.

In the revised manuscript, we included new analysis from Drop-seq with data properties that include deeper sequencing depth and a new platform different than 10X. In order to reflect differences in read counts, number of genes captured per cell, UMI base pair length, and amplification noise properties, our new data analysis was specifically based off of a second droplet-based sequencing platform that uses 3’ end counting technology, *viz.,* Drop-seq.

Reviewer #3: The paper systematically compares four dispersion metrics for scRNA-seq—Gini index, variance-to-mean ratio (VMR), variance, and Shannon entropy—via simulations across several distributions (Poisson, negative binomial, beta-Poisson, uniform), then applies the preferred metric to murine cardiomyocyte datasets (maternal hyperglycemia at E9.5/E11.5; and a trisomy-21 model). The study is well-structured and the analyses are compelling. however, minor revisions are needed before acceptance.

Thank you for your helpful feedback and consideration of our work.

- The manuscript uses “scale-invariant” to mean independence from dataset size, which is well supported; however, VMR = variance/mean can still inherit a mean-dependence for many count distributions (e.g., NB), so the text should clarify what kind of scale-invariance is claimed (dataset size vs. expression-level scaling) and delineate conditions where VMR may still drift with mean.

The reviewer raises a good point as scale invariance can have context dependent meaning. Thus, we have now provided context clarification in the revised manuscript. We now refer to this property as “independent of dataset size” where applicable. For example, on page 10 we refer to the following: “Scale invariance is an important property for quantifying transcriptional variability as transcriptional variability should be theoretically independent of dataset size.”

- Methods indicate VMR was computed on normalized, scaled counts per time point/condition. This can distort count-level mean–variance relationships and complicate interpretation. Please recompute VMR on raw UMI counts after size-factor normalization (or Pearson residuals) without per-gene scaling; and show concordance between “raw-VMR” and “scaled-VMR” rankings; and (iii) discuss implications.

The noise metrics were calculated on library-normalized, but not scaled, counts. Z-scaling the counts prohibits the use of the Gini index to quantify noise since the Gini index can only be applied to positive values. Additionally, as many dispersion metrics are based on the variance of gene expression, scaling and centering counts to fit the standard normal distribution results in loss of information about specific distributions and the underlying dispersion. For example, the Fano factor is the variance divided by the standard deviation. Applying a scaling transformation to the data changes the mean of every gene to approximately 0 and the variance of every gene to approximately 1. As a result, Fano factor values would be very large and difficult to interpret.

To validate this further, we calculated the Fano factor on raw UMI counts, normalized UMI counts, and scaled UMI counts of neurons from a murine dataset (PMID: 29281841). Here, normalized refers to library size normalization. Scaled refers to a Z-transform. We found a strong correlation between the Fano factor of genes calculated from the raw UMI counts and the normalized UMI counts. However, when we scaled the normalized counts using a Z-transform, we found no correlation between the Fano factor on the scaled counts and on the normalized counts, as expected. These results demonstrate that linear transformations have little effect on dispersion, but fixing the variance of a dispersion has an undesirable impact on dispersion.

**Impact of normalization and scaling on VMR.** (A) Comparison of VMR calculated on raw UMI counts versus on library-normalized UMI counts from mouse neurons at E11.5 (PMID:29281841). (B) Comparison of VMR calculated on library-normalized UMI counts versus on library-normalized and Z-transformed UMI counts.

- You note that correlations between VMR change and gene features depend on the threshold used to filter lowly expressed genes. Include a sensitivity analysis (multiple thresholds) and report stability metrics for top VMR-change genes and enriched pathways.

Thank you for raising this important point. To address this concern, we tested three thresholds for filtering genes that are expressed in low numbers of cells. Currently, to be kept in downstream analyses, genes must be expressed in at least 10 cells. In addition, we tested minimum cell cutoffs of 5 and 20 cells. After filtering out lowly expressed genes, we calculated the VMR of each gene at E11.5 and at E17.5 during neuronal differentiation using a Drop-seq data set (PMID: 29281841) and then ranked genes by the absolute change in VMR between the timepoints.

We compared the top 100 genes by absolute change in VMR from each filtering threshold and found that 75 genes (60.5%) were in the top 100 VMR genes regardless of which cutoff was used for filtering lowly expressed genes. We also found a very strong correlation between the rank of genes by absolute change in VMR calculated on data that excluded genes expressed in fewer than 5 cells and on data that excluded genes expressed in fewer than 10 cells. When we compared the rank of genes by absolute change in VMR calculated on data that excluded genes expressed in fewer than 10 cells versus on data that excluded genes expressed in fewer than 20 cells, we found a strong correlation. These results suggest that there is relative stability in VMR rankings, which provides some flexibility in the cutoff choice for the minimum number of cells a gene is expressed in.

**Gene rankings by change in VMR is consistent across different thresholds for removing lowly expressed genes.** (A) Venn diagram of the 100 genes with largest absolute change in VMR between E11.5 and E17.5 among neurons from data that excluded genes expressed in less than 5, 10, and 20 cells respectively. (B) Rank of genes by absolute change in VMR between E11.5 and E17.5 is similar between data that excludes genes expressed in less than 5 versus less than 10 cells. (C) Greater variation in rank of genes by absolute change in VMR appears when comparing data that excludes genes expressed in less than 10 versus less than 20 genes.

-Simulations span Poisson/NB/beta-Poisson/uniform, but common scRNA-seq nuisances (zero inflation/hurdle behavior, ambient RNA, library-size heterogeneity) could be reflected more explicitly. Consider adding simulations with spike-in technical noise or a hurdle-NB to test robustness of metric rankings.

Thank you for this suggestion. We have now added simulations with the hurdle-NB distribution and added these new data in the Methods and Results sections.

Briefly, we simulated counts from a hurdle negative binomial distribution. We tested two probabilities of seeing a non-zero count: 0.1 and 0.9. For each probability of a non-zero count, or hurdle parameter, the dispersion in the negative binomial component of the distribution was varied via the size parameter. One hundred size parameters were tested, and they ranged from 0.06 to 10. The theoretical variance between each instance of the hurdle negative binomial increased at a fixed exponential rate. For each instance of the hurdle negative binomial (ie, a given hurdle and size parameter), fifty counts matrices were simulated with varying numbers of cells. The smallest counts matrices contained 50 cells, and then largest counts matrices contained 5400 cells.

We observed that while all the metrics scaled well with the theoretical variance, entropy and Gini index were more robust and with a tighter distribution of noise estimates when the proportion of zeroes was 90% (hurdle-NB probability = 0.1) and became less robust as the proportion of zeroes decreased. On the other hand, VMR, CV, and CV^2^ appeared to be relatively stable in both hurdle-NB scenarios, suggestive of their advantageous properties for zero-inflated data. This new analysis has been included in S7 Fig and on page 12 of the manuscript:

“To further assess a common scRNA-seq feature – zero inflation – we also tested our metrics on hurdle-negative binomial (hurdle-NB) distributions. We simulated counts from a hurdle negative binomial distribution as described in the Methods. We observed that while all the metrics scaled well with the theoretical variance, entropy and Gini index were more robust and with a tighter distribution of noise estimates when the proportion of zeroes was 90% (hurdle-NB probability = 0.1) and became less robust as the proportion of zeroes decreased. On the other hand, Fano factor, CV, and CV^2^ appeared to be relatively stable in both hurdle-NB scenarios (S7 Fig), suggestive of their advantageous properties for zero-inflated data.”

**S7 Fig. Relative sensitivity of dispersion metrics to increasing transcriptional variability as modeled by the hurdle negative binomial distribution.** Heatmaps of each metric applied to simulated counts drawn from instances of the hurdle negative binomial distribution with hurdle parameters, which is the probability of a non-zero count, of 0.1 (A) and of 0.9 (D). The dispersion in the sampling distributions increases across the y-axis, as determined by the size parameter. The size of the simulated data increases across the x-axis, as determined by the number of genes in each counts matrix. (B, E) Heatmaps of relative change in each metric applied to counts from (A, D). (C, F) Histograms of deviation of relative change from mean relative change for each metric as calculated from the distributions described in (A, D).

-Because VMR corresponds to the classic Fano factor in count data, please make that synonym explicit, and consider adding comparisons to CV² (variance/mean²) and to variance-stabilized residual approaches to underscore when VMR is preferable. The current comparison to “variance” and “entropy” is useful but could miss widely used dispersion baselines.

Thank you for raising this important point. We have now added CV and CV² as two additional metrics as shown in Fig 2. We have also added the hurdle negative binomial and Poisson-lognormal distributions to encompass a broader range of data distributions. While these metrics may have utility in limited niche data distributions (e.g., overdispersed data where Entropy captures a broader dispersion), VMR remains a robust metric across our simulations for most data scenarios and is scale-invariant to dataset size. We have made the Fano factor synonym explicit, and added additional text of the Discussion reflecting this point:

“Of note, our findings may also have implications for feature selection approaches in scRNA-seq analysis. Several existing methods developed for feature selection are based on VMR, and this step in turn affects downstream analysis tasks such as cell type identification and clustering (PMID:37598422, PMID:40082610). Previous work has shown that highly variable genes influence the quality of scRNA-seq data integration, and our results imply that VMR would likely be a more robust choice for selecting these features in future projects, particularly those aimed at data integration across large cell atlas projects. VMR has also been used successfully to assess gene-level variability with biological implications in the context of innate immunity (PMID:32918862, PMID:37441161). We anticipate that variable genes are context-dependent, and the magnitude of dispersion will likely be different across scenarios.”

-You analyze matHG (E9.5/E11.5) and a T21 model. It would be helpful to quantify cross-timepoint and cross-dataset reproducibility of VMR-change rankings (e.g., rank-rank hypergeometric overlap, bootstrap stability). Also report how many cardiomyocytes per group underpin these estimates to contextualize variance in VMR itself.

In the matHG dataset, we identified 3502 cardiomyocytes, with 760 of those belonging to the control E9.5 condition, 827 to the matHG E9.5 condition, 752 to the control E11.5 condition, and 1163 to the matHG E11.5 condition. In the T21 dataset, we identified 830 cardiomyocytes across the T21 and control conditions. We appreciate the suggestion to analyze cross-timepoint and cross-dataset reproducibility, however, we have no *a priori* expectation of what these VMR-change rankings would look like. Moreover, comparing absolute VMR values across datasets/modalities is not as straightforward because it requires putting the different data on the same scale. This idea is an interesting point that will certainly be an area of investigation for future work but remains outside the scope of our current manuscript.

-For the “top-k” overlaps (100/1000), specify how significance/ranking for DEGs and VMR-change sets were controlled (e.g., FDR for DEG list; whether any p-values or empirical nulls were used for VMR changes). Including q-value cutoffs and enrichment NES/FDRs will add rigor.

For KEGG GSEA, Benjamini-Hochberg was used for multiple testing correction, and the adjusted p-value cutoff was 0.05. DEGs were ranked by adjusted p-values and had an FDR of ≤ 20%. These details have now been added to Methods.

- For KEGG GSEA and RcisTarget, please describe background gene sets, species/annotation versions, and multiple-testing thresholds; given the non-overlap of KEGG pathways between DE and VMR lists, a permutation-based concordance test (e.g., gene-set co-enrichment) would be informative.

For KEGG GSEA, the background gene set was all genes in the mouse genome from [org.Mm.eg.db](http://org.mm.eg.db) v3.18, which is based on Entrez Gene identifiers. Benjamini-Hochberg was used for multiple testing correction, and the adjusted p-value cutoff was 0.05.

For RcisTarget, the background for motif enrichment was a database of gene-motif rankings generated from GRCm38 (mm10) that included 10 kbp upstream and downstream of each gene (PMID:28991892). Motif enrichment analysis returns normalized enrichment score (NES).

These details are included in the Methods section.
