# Supplementary material for: Assessment of dispersion metrics for estimating single-cell transcriptional variability
Source: PLoS Comput Biol. 2026 Mar 2;22(3):e1014030. doi: 10.1371/journal.pcbi.1014030 (PMC12970974; doi:10.1371/journal.pcbi.1014030)
Supplement: Attachment — Submitted filename: Response_to_Reviewers_auresp_2.docx [file pcbi.1014030.s016.docx]

**PCOMPBIOL-D-25-01049R1**

We appreciate that reviewers agree our revisions have greatly improved the manuscript. Please find our response addressing the remaining comments from Reviewer 2 below.

**Reviewer 2.**

Re: The discussion of the extent of biological insight, specifically, the interpretation of the fact VMR and DEG analyses yield different sets of genes.

I suggest that the authors further examine the expression patterns of the genes showing changes in VMR.

In particular, it would be informative to determine whether the observed VMR changes reflect shifts in the fraction of expressing cells across the population, or instead correspond to 'analogue' changes in expression levels within individual cells. Such an analysis would align well with the authors’ discussion of this question and provide additional biological interpretation of the nature of VMR-associated genes (in comparison to DEGs).

To address this point, we compared the observed changes in VMR to both change in fraction of non-zero UMI expressing cells and change in mean expression in a Drop-seq dataset of murine neurons at E11.5 and at E17. We found a negative correlation between change in VMR and both of these variables, suggesting that there is an overlap in the information captured by VMR, mean expression, fraction of expressing cells. However, the degree of correlation varied between different datasets and sequencing platforms, suggesting that VMR may capture information different from fraction of expressing cells, such as ‘analogue’ changes. To further explore this idea, we examined the distribution of UMI counts between timepoints. Some genes, such as *Neurod6*, displayed a large shift in fraction of expressing cells. Others, like *Stmn2*, had a relatively smaller change in fraction of expressing cells, and displayed a change in the shape of the distribution of counts. *Neurod6* had the largest absolute change in VMR between timepoints, and *Stmn2* was among the top 20 genes with largest absolute change in VMR. *Neurod6* is a DEG, while *Stmn2* is not.

We performed similar analysis on the E9.5 timepoint of a cardiomyocyte 10x Genomics dataset. Change in VMR between control and maternal hyperglycemia (matHG) conditions inversely correlated with change in fraction of expressing cells and change in mean expression. Upon examination of the top genes with largest absolute change in VMR, we similarly found both genes that displayed a large change in fraction of expressing cells, like *Hbb-bt*, and genes that displayed a large change in shape of UMI count distribution, like *Igfbp5*. Of note, *Igfbp5* encodes insulin-like growth factor binding protein 5, which has been implicated in the progression of gestational diabetes mellitus in humans (PMID:26067361). Similar analysis was performed on another cardiomyocyte 10x Genomics dataset comparing WT and T21 mice (PMID:38266108), and again we found negative correlation between change in VMR, change in fraction of expressing cells, and change in mean expression. Collectively, our analyses suggest that transcriptional variability can be a useful tool for investigating underlying biological mechanisms irrespective of the sequencing technology.

We have added new text and an additional Fig 6 to address this point.

**Fig 6. Change in VMR has a negative correlation with mean and fraction of expressing cells and varies between platforms.** In a Drop-seq neuron dataset, change in VMR inversely correlates with (A) the fraction of expressing cells and (B) change in mean expression. (C) *Neurod6,* a DEG, is the gene in this dataset with the largest absolute change in VMR between E11.5 and E17.5, whereas *Stmn2,* a non-DEG, also had a large absolute change in VMR between timepoints. In the 10x Genomics dataset comparing cardiomyocytes in control and matHG conditions at E9.5, associations between VMR and UMI counts across cells behave similarly (D, E). Panel (F) shows *Hbb-bt,* a DEG with the largest absolute change in VMR at E9.5 between control and matHG. *Igfbp5* had the 10th largest absolute change in VMR between conditions, and is not a DEG. A negative correlation between VMR changes and UMI distribution is again evident in the 10x Genomics dataset comparing WT and T21 cardiomyocytes (G, H). Panel (I) shows *Thbs1*, a gene with the largest absolute change in VMR between WT and T21 conditions. *Col2a1* had the 10th largest absolute change in VMR between conditions, and had the largest fraction of expressing cells among the top 10 genes with largest change in VMR. Neither *Thbs1* nor *Col2a1* are DEGs.

Fig S10B, please provide consistent formatting for p-values (i.e., number of digits denoted)

Formatting of p-values is now consistent throughout the manuscript.

Fig. 5C: “We found that enriched TF motifs differed between the two groups (Fig 5C, S1 Table). For example, distinct motifs found among the genes with largest change in VMR include Tead, Fos, Jun, and Ctcf.”
For clarity please label different motifs referred to in C (and presumably S10). What are the key motifs corresponding to DEGs as presented in the panel?

We have labeled motifs in Fig 5C and S10 Fig according to the reviewer's suggestion.
